# A study protocol for HEalth-Related quality of life-intervention in survivors of Breast and other cancers experiencing cancer-related fatigue using TraditionAL Chinese Medicine: the HERBAL trial

**DOI:** 10.1186/s13063-020-04810-4

**Published:** 2020-11-04

**Authors:** Ning Yi Yap, Wei Sheng Loo, Huang Fang Zheng, Quan Ming Tan, Tze Kiat Tan, Leona Yan Peng Quek, Chia Jie Tan, Yi Long Toh, Chiu Chin Ng, Seng Kok Ang, Veronique Kiak Mien Tan, Han Kiat Ho, Lita Chew, Kiley Wei-Jen Loh, Tira Jing Ying Tan, Alexandre Chan

**Affiliations:** 1 Department of Pharmacy, National University of Singapore, Singapore, Singapore; 2 Department of Pharmacy, National Cancer Centre Singapore, Singapore, Singapore; 3 Singapore Thong Chai Medical Institution, Singapore, Singapore; 4 Division of Surgery & Surgical Oncology, National Cancer Centre Singapore, Singapore, Singapore; 5 Division of Medical Oncology, National Cancer Centre Singapore, Singapore, Singapore; 6 Department of Clinical Pharmacy Practice, University of California, Irvine, USA

**Keywords:** Randomized controlled trial, Cancer survivors, Quality of life, Cancer-related fatigue, Traditional Chinese medicine

## Abstract

**Background:**

Cancer-related fatigue (CRF) is a debilitating condition which commonly affects cancer survivors. The management of CRF remains a challenge due to the lack of effective pharmacological interventions. Traditional Chinese medicine (TCM) could be a potential therapeutic option for CRF. The modified Xiang Bei Yang Rong Tang (XBYRT) is a TCM herbal decoction, formulated to improve fatigue symptoms in cancer survivors. This clinical trial aims to evaluate the efficacy and safety of XBYRT in improving CRF and quality of life (QOL) of cancer survivors.

**Methods:**

This is a single centre, randomized, double-blind, placebo-controlled, parallel trial. Eighty cancer survivors will be recruited and randomized to receive the XBYRT or placebo decoction, in a ratio of 1:1. Participants will consume the XBYRT/placebo decoction daily for 8 weeks and undergo assessments at baseline and 4, 8 and 10 weeks after baseline. The participants will be assessed for patient-reported outcomes (PRO), blood biomarkers and adverse events at each time point. The primary outcome is the overall health and QOL status, at 8 weeks follow-up. The secondary outcomes are the effects of XBYRT on fatigue levels, cancer-related cognitive impairment and QOL, as assessed by PRO. The incidence of adverse events and the effects of the XBYRT decoction on blood biomarkers associated with CRF will also be evaluated.

**Discussion:**

Efficacy and safety outcomes from this trial will provide important clinical data to guide future large-scale randomized controlled trials, and the evaluation of the objective blood biomarkers can help to delineate the biological mechanisms of CRF.

**Trial registration number:**

ClinicalTrials.govNCT04104113. Registered on 26 September 2019

## Introduction

Advances in diagnosis and treatment have resulted in better survival rates and an increase in the number of cancer survivors [1]. Cancer survivors often experience a wide range of radiotherapy or chemotherapy-induced side effects which may persist for years even after the cessation of treatment. Cancer-related fatigue (CRF) has been reported as the most distressing symptom experienced by cancer patients and can have debilitating physical, emotional and cognitive effects which negatively impact the patients’ quality of life (QOL) [2, 3]. Up to 45% of cancer patients suffer from clinically significant CRF during treatment, and approximately 29% still experience fatigue after the completion of treatment [4].

The pathophysiology and mechanisms leading to CRF and the factors causing persistent fatigue in cancer survivors are not well understood, despite it being a highly prevalent symptom in cancer patients and survivors. This is because the pathophysiology underlying CRF is complex involving psychological, physical and biological factors. One of the postulated biological factors contributing to the development of CRF is inflammation from immune activation caused by cancer and its treatment. Dysregulation of circulating inflammatory markers and cytokines such as interleukin-1 receptor antagonist (IL-1RA), interleukin-6 (IL-6) and C-reactive protein (CRP) has been reported in cancer patients and survivors experiencing CRF [5–8]. Chemotherapy, along with a pro-inflammatory environment, induces a number of downstream toxic effects, including an increase in reactive oxygen species which can result in mitochondrial impairment [9–11]. Mitochondrial dysfunction has been implicated as one of the possible biological factors of chronic fatigue syndrome and CRF [12–14].

In Asia, many cancer patients seek complementary and alternative medicine (CAM) to manage their cancer-related symptoms or improve their general health, and one of the most commonly used CAM is traditional Chinese medicine (TCM) [15–17]. According to the TCM concept, CRF is characterized by the deficiency of qi, the vital energy of the body, along with blood deficiency [18]. Chemotherapy and radiotherapy deplete both qi and blood by indiscriminately destroying growing cells, damaging the bone marrow and weakening the body. Qi deficiency was found to be associated with CRF and a poorer QOL in cancer patients [18, 19]. In addition, a few studies looking at various TCM decoctions have shown effectiveness in improving fatigue levels in cancer patients [20–23]. However, the efficacy of TCM is not backed with robust clinical evidence and safety data as most of these studies suffer from the lack of adequate control and blinding which might introduce bias in the results. Therefore, more well-designed randomized controlled clinical trials are needed to evaluate the efficacy and safety of TCM in treating CRF.

The TCM formula for this randomized controlled trial is a modification of Xiang Bei Yang Rong Tang (香贝养荣汤). Xiang Bei Yang Rong Tang (XBYRT) was described in the ancient TCM text “Yi Zong Jin Jian” volume 64 and is used for nourishing qi and blood [24]. The 15 herbal components in the modified XBYRT formula were selected based on their ability to augment qi, nourish the blood, improve appetite and calm the mind which can help to alleviate CRF symptoms in cancer survivors.

The primary objective of this trial is to evaluate the efficacy of XBYRT in improving the overall health status and QOL in a cohort of cancer survivors, assessed using the global health status of the European Organization for Research and Treatment of Cancer Quality of Life Questionnaire (EORTC QLQ-C30), at 8 weeks follow-up. The secondary objectives of this trial are to determine the effects of XBYRT on fatigue levels, cancer-related cognitive impairment and QOL. In addition, the safety outcomes associated with XBYRT and the impact of the TCM decoction on biomarkers associated with CRF will be evaluated.

## Methods

### Study design

This is a single-centre randomized, double-blind, placebo-controlled, parallel trial, and the protocol for this trial has been prepared in accordance with the Standard Protocol Items: Recommendations for Interventional Trials (SPIRIT)–TCM extension statement 2018 (Additional file 1) [25]. This study protocol has received ethical approval from the Centralised Institutional Review Board (2019/2135) and is registered in ClinicalTrials.gov (NCT04104113). Written informed consent will be obtained from all study participants. A total of 80 cancer survivors will be enrolled from the outpatient oncology clinics at the National Cancer Centre Singapore (NCCS), and the recruitment and follow-up period for this trial will be from October 2019 to December 2021.

### Participants

The target study participants are cancer survivors who complain of fatigue and will be recruited through referrals from oncologists. Cancer survivors will be screened for fatigue using a single item, where patients will be asked to rate their fatigue on a scale of 0 to 10 over the past 7 days. A score of 0 means no fatigue, and mild fatigue is indicated as a score of 1 to 3, moderate fatigue as 4 to 6 and severe fatigue as 7 to 10 [26]. Patients with ratings ≥ 4 are considered as experiencing significant fatigue. Potential participants will also be screened by certified TCM physicians based on TCM syndrome differentiation. The eligibility criteria are as follows:

1. Age ≥ 21 years
2. Clinically diagnosed cancer (stages I–III)
3. Completed surgery/chemotherapy/radiotherapy for at least 1 month
4. At least 1 month after starting on aromatase inhibitors or ovarian suppression for breast cancer survivors
5. Not expected to receive surgery/chemotherapy/radiotherapy for the next 10 weeks
6. Fatigue screening score ≥ 4 for the past 7 days
7. Life expectancy ≥ 3 months
8. Patients who satisfy TCM syndrome differentiation as qi and blood deficiency: experience 2 major symptoms coupled with typical tongue and pulse conditions; 2 major symptoms and 1 possible symptom coupled with tongue and pulse conditions; and 1 major symptom and at least 2 possible symptoms coupled with tongue and pulse conditions
9. Able to read and understand English or Mandarin

The exclusion criteria are as follows:

1. Cancer recurrence and/or metastasis.
2. Untreated co-morbidities causing fatigue (e.g. severe anaemia, thyroid disorder).
3. On medications that cause fatigue (e.g. beta blockers).
4. Patients on warfarin.
5. Cancer survivors receiving adjuvant therapy during the study period. Aromatase inhibitors and anti-human epidermal growth factor receptor 2 (HER2) monoclonal antibodies are acceptable.
6. Receiving or planning to receive treatment from other TCM practitioners during the study period.
7. Breastfeeding or intending to conceive/get pregnant during the study treatment period.
8. Patients who present with yin deficiency and excess syndromes (e.g. phlegm-dampness, blood stasis, toxic heat and qi stagnation).

### Intervention

Study participants will take either the assigned XBYRT or placebo which is prepared as granules once daily for a duration of 8 weeks. Participants are required to dissolve the XBYRT or placebo granules in a cup of hot water and consume the decoction. During the study period, participants will be asked to refrain from taking other supplements or herbal medicines.

The experimental group will be given the XBYRT granules. The formulation, the TCM indication and the daily dosage of the XBYRT are displayed in Table 1. The study dosage is selected based on the safety and efficacy guidelines from the *Pharmacopoeia of the People’s Republic of China* and *General Eleventh Five-Year National Planning Textbook for Higher Education: Chinese Materia Medica* [27, 28]. The dosage indicated in Table 1 represents the amount of raw materials required for the extraction of the granules. The dried granules are extracted from the boiled aqueous concentrate of the raw materials. The final daily dosage consists of 24 g of extracted granules.

**Table 1:** Formula and components of the modified Xiang Bei Yang Rong Tang decoction

Chinese name	Chinese name (Pinyin)	Scientific name	Dosage (g)	Purported effect
黄芪	Huang Qi	Radix Astragaliseu Hedysari	15	Augments qi and raises yang, augments defensive qi, consolidates the superficies, promotes drainage of pus and healing, facilitates water movement and reduces swelling
党参	Dang Shen	Radix Codonopsis Pilosulae	15	Tonifies the middle jiao, augments qi and generates fluids and blood
白术	Bai zhu	Rhizoma Atractylodis Macrocephalae	12	Augments qi, strengthens the spleen, dries dampness, promotes diuresis and stops sweating
茯苓	Fu Ling	Poria	15	Drains water, dissipates dampness, strengthens the spleen and calms the mind
白芍	Bai Shao	Radix Paeoniae Alba	15	Nourishes the blood, retains yin, soothes the liver and relieves pain, and stops excessive perspiration
枸杞子	Gou Qi zi	Fructus Lycii	12	Nourishes the liver and kidney, clears the eyes and moistens the lung
女贞子	Nü Zhen Zi	Fructus Ligustri Lucidi	12	Tonifies the liver and kidney, cools heat and clears the eye
车前子	Che Qian Zi	*Plantago asiatica*	12	Induces diuresis, drain dampness, improve vision and resolve phlegm
鸡内金	Ji Nei Jin	Endothelium Corneum Gigeriae Galli	10	Promotes digestion and invigorate spleen, arrest seminal emission and relieve enuresis
生麦芽	Sheng Mai Ya	*Hordeum vulgare* L.	15	Promotes digestion and invigorate spleen, stop lactation and release distension
益智仁	Yi Zhi Ren	Fructus Alpinia oxyphylla	10	Tonifies kidney yang, secure essence and reduce urination, warm spleen yang, improve appetite and reduce salivation
香附	Xiang Fu	Rhizoma Cyperi	10	Unblocks the liver and regulates qi, regulates menstruation and stops pain
远志	Yuan Zhi	Radix Polygalae	10	Stabilizes the heart and calms the mind, dissolves phlegm and opens orifices, and reduces abscesses and swelling
浙贝母	Zhe Bei Mu	Bulbus Fritillariae Thunbergii	10	Clears heat and resolves phlegm, disperses masses/abnormal growth and promotes the healing of carbuncles
土茯苓	Tu Fu Ling	Smilax glabra Roxb	15	Removes toxicity, excrete dampness and ease joint movement

The control group will receive a daily dosage containing 24 g of placebo granules. The placebo granules comprise of 5% of the herbal components, 95% maltodextrin, colourant and 0.002% denatonium benzoate as bitterant. This is to ensure that the placebo possesses the taste and smell of the herbal components. For this reason, it is a common practice in TCM trials to incorporate herbal components ranging from 5 to 10% in the placebo decoction, and this concentration is unlikely to exert a therapeutic effect [29, 30]. The placebo will be colour adjusted to match the XBYRT granules, and the packaging for the XBYRT and placebo granules will be similar in appearance. In order to encourage adherence to the study intervention, participants will be reminded to take their daily dosages and record their missed dosages.

Both the XBYRT and placebo granules will be manufactured by Kinhong Pte Ltd., Singapore, a Good Manufacturing Practices-certified manufacturer. A certificate of analysis containing the results on component identification, moisture content and the absence of heavy metal and microbial contamination will be available for each batch of XBYRT/placebo granules manufactured.

### Randomization, allocation concealment and blinding

Recruited participants will be randomized into the XBYRT or placebo treatment arm by block randomization at a 1:1 ratio, with a block size of 10. The consultant physicians, study team, trial pharmacists and study participants are blinded to the intervention to minimize potential bias. Randomization and blinding will be performed by a third party clinical trial service provider, Singapore Clinical Research Institute, using the sealed envelope method. Participants will collect the granules from the clinical trial pharmacy at NCCS based on their pre-assigned numbers.

### Sample size

In order to ensure that the sample size is adequately powered to estimate a reliable standard deviation (SD) for a phase III trial, Teare et al. has recommended to include at least 70 subjects (35 per arm) for estimating the SD for a continuous outcome [31]. Therefore, to account for a 10% dropout, a total sample size of 80 patients (rounded up from 78), with 40 participants on each arm, is required for this study.

### Assessments

**Fig. 1:**
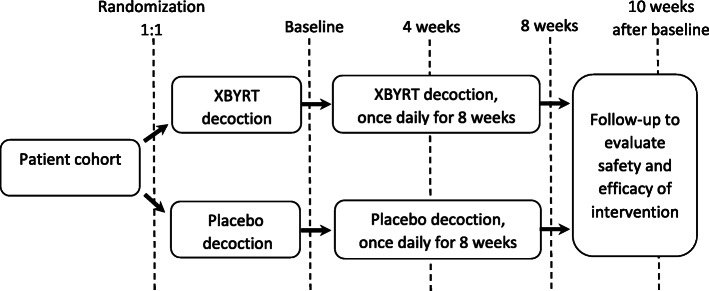
Flow diagram of the study design and assessment time points

**Fig. 2:**
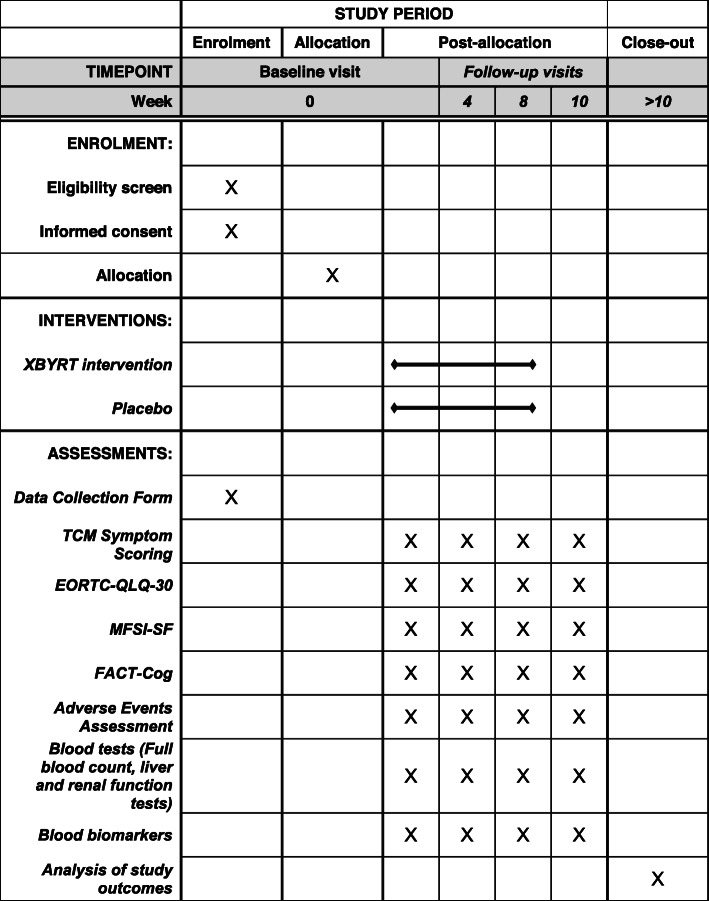
SPIRIT figure for the enrolment, interventions and assessments of the study

Participants will be assessed at four time points: baseline and 4 weeks, 8 weeks and 10 weeks after baseline (Fig. 1). At the baseline visit, the participant’s demographics, medical history, cancer diagnosis and concomitant medications will be recorded in a standardized data collection form. The participants will be assessed for patient-reported outcomes (PRO) and objective blood biomarkers at each time point (Fig. 2). Participants will also be monitored for safety and adverse events (AE).

#### Assessment of quality of life

The European Organisation for Research and Treatment of Cancer Quality of Life Questionnaire (EORTC-QLQ 30) is a self-administered questionnaire that measures the health-related QOL. The English and Chinese versions of this questionnaire have been validated in Singaporean cancer patients [32, 33]. It consists of 30 items across five functional domains (physical, role, cognitive, emotional and social), three symptom domains (fatigue, pain and nausea/vomiting), six individual items (dyspnea, insomnia, anorexia, diarrhoea, constipation and financial stability) and a global health status domain. All items are linearly transformed into a score ranging from 0 to 100. A higher score represents better functional status and health-related QOL in the functional and global health status domain while a higher score in the symptom domain or individual symptom item is interpreted as a worse extent of symptoms experienced by the patient.

#### Assessment of fatigue

The Multidimensional Fatigue Symptom Inventory-Short Form (MFSI-SF) is a multidimensional questionnaire for measuring fatigue in cancer patients. The English and Chinese versions of MFSI-SF have been validated in breast cancer patients in Singapore [34]. This 30-item questionnaire contains five subscales: general fatigue, physical fatigue, emotional fatigue, mental fatigue and vigour. Specific subscale scores are obtained by tabulating the item scores from each subscale. The MFSI-SF subscale scores can be combined to obtain an overall score. A higher score reflects a worse fatigue level.

#### Assessment of cognition

The Functional Assessment of Cancer Therapy-Cognitive Function (FACT-Cog) version 3 is used to assess subjective cognitive function. Both the English and Chinese versions of FACT-Cog have been validated in cancer patients in Singapore [35]. This questionnaire has 37 items containing assessments for six domains of cognition (memory, concentration, mental acuity, verbal ability, functional interference and multitasking) and two subscales for noticeability and impact on QOL. A global FACT-Cog score is derived from the total of the domain/subscale scores. Higher scores indicate better perceived cognitive abilities and QOL.

#### TCM symptom scoring

The TCM symptom scoring form is referenced from the combination of qi and blood deficiency from the National Standards of People Republic of China and details the method of pattern identification [36]. The form contains assessments for 9 major TCM symptoms and 7 possible TCM symptoms (Table 2). These symptoms are scored on an ordinal scale of absent, mild, moderate and severe. The scoring tool will be completed by the assessing TCM physician, and the patient’s pulse and tongue appearance will be assessed from the TCM’s perspective. Information collected from the questionnaire will be used to screen for inclusion and monitor the response of the participants towards the TCM decoction.

**Table 2:** Major and possible symptoms assessed for TCM symptom scoring

Major symptoms	Possible symptoms
Shortness of breath	Spontaneous sweating
General fatigue	Reluctance to talk
Weakness	Insomnia
Pale or sallow complexion	Limb numbness
Dizziness, blurred vision	Scanty menstruation
Heart palpitation	Nausea, vomiting
Poor appetite	Weakness in defaecation
Abdominal distension after food	
Loose stools	

### Safety monitoring

Safety of study participants will be monitored at every time point assessment through blood tests evaluating organ functions such as liver and renal function tests, electrolyte level and full blood count. Participants will be asked to report any adverse effects experienced during the follow-up period which are graded according to the Common Terminology Criteria for Adverse Events (CTCAE) version 5 [37]. There are five grades in ascending order of severity, with grade 1 representing mild symptoms and grade 5 representing death. In order to monitor the adverse events (AEs) or serious adverse events (SAEs), an independent Data and Safety Monitoring Board (DSMB) review will convene for every 20 patients recruited for the trial. Therefore, there will be a total of three DSMB meetings for this trial. Analysis comparing the incidence of AEs based on the intervention group will be conducted at each DSMB review. The DSMB will make recommendations to the study team regarding the appropriate courses of action to address study safety issues which may arise during the trial. Unblinding to the study team is allowed only when the identification of the intervention arm is absolutely necessary for further management of the participant, for example, in the event of a SAE.

### Assessment of biomarkers

Blood samples from participants will be collected for biomarker assessments at all assessment time points.

#### Mitochondrial DNA content

The mitochondrial DNA (mtDNA) will be extracted from the buffy coat, and mtDNA content will be evaluated in relation to CRF. To measure mtDNA content, real-time quantitative polymerase chain reaction (PCR) will be performed using the Quantifast SYBR Green Master Mix from Qiagen along with the forward and reverse primers for Mito and β2 microglobulin (B2M). The Mito primer pair will amplify the ZFP28 zinc finger protein gene in mtDNA, while the B2M primer pair targets the beta-2-microglobulin gene in nuclear DNA (nDNA). The analysis will be performed in triplicates on 96-well plates, and target sequences will be amplified in a Biorad CFX96 Touch cycler. The average threshold cycle (Ct) values of nuclear DNA and mtDNA from each triplicate series are obtained, and the ΔCt method is applied (ΔCt = CtnDNA − CtmtDNA). The relative mtDNA copy number is derived from 2 × 2^ΔCt^, taking into account that nDNA is diploid.

#### Inflammatory cytokines

Plasma levels of inflammatory marker C-reactive protein (CRP) and cytokines TNF-α, IL-1β, IL-6 and IL-8 will be measured. The choice of cytokines is based on the findings of a systematic review which suggest that the development of CRF is influenced by immune dysregulation of which the aforementioned cytokines have been shown to contribute to worsening or persistent fatigue [8]. The cytokines of interest will be quantified using the highly sensitive multiplex immunoassay (Luminex®).

#### Oxidative stress markers

Plasma levels of malondialdehyde (MDA), superoxide dismutase (SOD) and glutathione peroxidase (GSH-Px) will be measured using commercially available assay kits.

### Outcomes

#### Primary outcome

The primary endpoint is the difference in global health status (GHS) score between the XBYRT interventional and placebo arms from baseline to 8 weeks after baseline. The GHS is a two-item domain from the EORTC-QLQ 30.

#### Secondary outcomes

Secondary outcomes are the changes in MFSI-SF and FACT-Cog total scores, including the subscale or domain scores between the XBYRT and placebo arms from baseline to 4, 8 and 10 weeks after baseline. Changes in the functional and symptom domain scoring of the EORTC QLQ-C30 from baseline will also be evaluated. Safety outcomes will be reported as the incidence of AEs or SAEs in study participants.

#### Biomarkers

Changes in circulating levels of inflammatory markers CRP, TNF-α, IL-1β, IL-6 and IL-8 from baseline to 4, 8 and 10 weeks after baseline will be compared between the XBYRT and placebo arms. Circulating oxidative stress markers, malondialdehyde (MDA), superoxide dismutase (SOD) and glutathione peroxidase (GPx), as well as mtDNA levels, will also be compared between the XBYRT and placebo arms for the aforementioned time points.

### Data management

The research data will be entered into a password-protected electronic database, and the entered data will be checked with the source documents to ensure data accuracy, by an independent study team member who is not in charge of data entry. The hard copy of study records or electronic study database will be kept in controlled access locations under lock and key. Investigators involved in this study will have access to the final dataset.

### Statistical analysis

The difference in GHS scores at 8 weeks after baseline between XBYRT intervention and placebo will be assessed using independent *t* test. To assess for the longitudinal effect of score changes over time, linear mixed models will be used to compare the outcomes between XBYRT intervention and placebo at the respective time points, adjusting for baseline values and clinically relevant factors. Individual-specific effects will be treated as random effects. Biomarker levels will also be evaluated using *t* tests and linear mixed models. The safety outcomes will be reported in proportions of patients experiencing AEs (% in terms of incidence) using the CTCAE v5.0 Criteria. Chi-square test will be used to evaluate the difference in severity (by grades) between the XBYRT intervention and placebo groups. An intention-to-treat (ITT) analysis approach will be adopted, and the data from all participants who are randomized and completed the trial without any major violation of the protocol will be analysed. Missing data will be treated as missing completely at random.

### Study dissemination

The findings from this trial will be disseminated through conference presentations and publications in peer-reviewed journals.

### Patient and public involvement

Patients or the public were not involved in the design, conduct or reporting of the research.

## Discussion

Currently, CRF is often under-reported and under-treated as there is a limited understanding of effective management strategies for the symptoms [38]. The current non-pharmacological recommendations for managing CRF include exercise, cognitive behavioural therapy and patient education [39, 40]. However, pharmacological therapies are still investigational and have limited efficacy [39, 40]. Hence, there is a need to gain a clearer understanding of CRF aetiology and identify therapies that could effectively manage CRF in cancer survivors.

TCM presents an attractive therapeutic option for the management of CRF symptoms as the concept of using TCM decoctions to replenish the qi or the body vital energy and improve sleep disturbances has been recorded in classical traditional Chinese medical texts [41]. The biological effects of TCM have been investigated in a number of in vitro and in vivo studies, and among the proposed mechanisms for the positive effects of herbal TCM on fatigue are the anti-inflammatory, immuno-modulatory and antioxidant abilities of the herbal components [41–43]. For example, supplementation with Radix Astragali extract enhanced endurance and reduced the levels of reactive oxygen species and cytokines in mice and rats with exercise-induced fatigue [42, 43]. One of the main TCM uses of Radix Paeoniae Alba is to treat blood deficiency, and an in vivo investigation has demonstrated that Radix Paeoniae Alba extract increased haemoglobin, haematocrit and serum erythropoietin in anaemia-induced rats [44]. Phytochemicals extracted from Radix Codonopsis Pilosulae, Rhizoma Atractylodis Macrocephalae, Poria and Fructus Lycii also exerted anti-inflammatory, immuno-modulatory and antioxidative activities in vitro and in vivo [45–48]. Therefore, the analysis of the cytokines, antioxidant markers and mtDNA levels of participants in the XBYRT intervention and placebo arms in this trial will determine the effects of the XBYRT decoction on the inflammatory and oxidative stress status of patients with CRF. Cytokines and mtDNA are also potential predictive biomarkers for CRF [5, 7, 14]. These markers are useful as they will provide an objective observation on the patients’ response to the TCM concoction.

Although in vitro and in vivo studies have shown evidence supporting the purported effects of these herbal medicines, there is still a dearth of clinical evidence due to the limited number of clinical trials investigating the use of herbal TCM in treating CRF. In a single-arm study, the use of Ren Shen Yangrong Tang (RSYRT) was investigated in 33 cancer survivors who have reported moderate to severe level of fatigue [20]. RSYRT, which contains 12 herbal components, was formulated to correct qi deficiency, and the formulation consists of some similar herbal ingredients to the XBYRT used in this protocol, such as the Radix Codonopsis, Rhizoma Atractylodis Macrocephalae, Radix Paeoniae Alba, Poria Cocos and Radix Polygalae. A significant decrease in fatigue severity was seen in patients after 6 weeks of treatment with RSYRT [20]. In a randomized controlled trial, 40 patients with CRF were randomized to receive either an intervention, Bu-Zhong-Yi-Qi-Tang (BZYQT) or no intervention. BZYQT contains 10 herbs with Radix Astragali, *Atractylodis lanceae* rhizome and Radix Ginseng as the main components [21]. Patients who received BZYQT for 2 weeks reported significant improvements in fatigue levels and QOL [21]. Although these studies reported positive outcomes, they were not adequately controlled to eliminate possible bias or placebo effects.

Therefore, the outcome from this randomized, double-blind, placebo-controlled clinical trial will contribute valuable clinical evidence on the efficacy and safety of a TCM decoction for managing CRF symptoms and QOL in a cohort of cancer survivors. Results obtained from this study will also generate important data for the design of a larger phase III clinical trial.

## Conclusion

In summary, this HERBAL study evaluates the efficacy of a modified TCM decoction, the XBYRT, which is specifically formulated for addressing qi and blood deficiency to manage CRF symptoms in cancer survivors. The results from this clinical trial can provide both clinical and biochemical basis and guidance for future large-scale randomized controlled trials and facilitate a better understanding of possible biological mechanisms contributing to the effects of TCM.

### Trial status

At the time of submission, recruitment is still ongoing. The recruitment and follow-up period for this trial will be from October 2019 to December 2021.

At manuscript submission, the study protocol used is the version 10 (15 October 2020), approved on 23 October 2020.

## Supplementary Information

**Additional file 1.** SPIRIT 2013 Checklist: Recommended items to address in a clinical trial protocol and related documents.

## Data Availability

This is an investigator-initiated trial; therefore, the investigators will have access to the trial dataset.

## Abbreviations

AE: Adverse event
B2M: β2 microglobulin
CAM: Complementary and alternative medicine
CRF: Cancer-related fatigue
CRP: C-reactive protein
CTCAE: Common Terminology Criteria for Adverse Events
DSMB: Data and Safety Monitoring Board
EORTC QLQ-C30: European Organization for Research and Treatment of Cancer Quality of Life Questionnaire
FACT-Cog: Functional Assessment of Cancer Therapy-Cognitive Function
GHS: Global health status
GSH-Px: Glutathione peroxidase
IL: Interleukin
MDA: Malondialdehyde
MFSI-SF: Multidimensional Fatigue Symptom Inventory-Short Form
mtDNA: Mitochondrial DNA
NCCS: National Cancer Centre Singapore
PCR: Polymerase chain reaction
PRO: Patient-reported outcomes
QOL: Quality of life
SAE: Serious adverse event
SD: Standard deviation
SOD: Superoxide dismutase
TCM: Traditional Chinese medicine
XBYRT: Xiang Bei Yang Rong Tang
